# Microbiota in vaginal health and pathogenesis of recurrent vulvovaginal infections: a critical review

**DOI:** 10.1186/s12941-020-0347-4

**Published:** 2020-01-28

**Authors:** Namarta Kalia, Jatinder Singh, Manpreet Kaur

**Affiliations:** 1 Department of Molecular Biology & Biochemistry, Guru Nanak Dev University, Amritsar, 143005 India; 2 Department of Human Genetics, Guru Nanak Dev University, Amritsar, 143005 India

**Keywords:** Bacteriobiota, Community state types (CSTs), Mixed infections, Mycobiota, Menopause, Non-*albicans Candida* (NAC) Species, Vaginal Mycobiome, Vaginal ecosystem and vaginal dysbiosis

## Abstract

Recurrent vulvovaginal infections (RVVI) has not only become an epidemiological and clinical problem but also include large social and psychological consequences. Understanding the mechanisms of both commensalism and pathogenesis are necessary for the development of efficient diagnosis and treatment strategies for these enigmatic vaginal infections. Through this review, an attempt has been made to analyze vaginal microbiota (VMB) from scratch and to provide an update on its current understanding in relation to health and common RVVI i.e. bacterial vaginosis, vulvovaginal candidiaisis and Trichomoniasis, making the present review first of its kind. For this, potentially relevant studies were retrieved from data sources and critical analysis of the literature was made. Though, culture-independent methods have greatly unfolded the mystery regarding vaginal bacterial microbiome, there are only a few studies regarding the composition and diversity of vaginal mycobiome and different *Trichomonas vaginalis* strains. This scenario suggests a need of further studies based on comparative genomics of RVVI pathogens to improve our perceptive of RVVI pathogenesis that is still not clear (Fig. 5). Besides this, the review details the rationale for *Lactobacilli* dominance and changes that occur in healthy VMB throughout a women’s life. Moreover, the list of possible agents continues to expand and new species recognised in both health and VVI are updated in this review. The review concludes with the controversies challenging the widely accepted dogma i.e. “VMB dominated with *Lactobacilli* is healthier than a diverse VMB”. These controversies, over the past decade, have complicated the definition of vaginal health and vaginal infections with no definite conclusion. Thus, further studies on newly recognised microbial agents may reveal answers to these controversies. Conversely, VMB of women could be an answer but it is not enough to just look at the microbiology. We have to look at the woman itself, as VMB which is fine for one woman may be troublesome for others. These differences in women’s response to the same VMB may be determined by a permutation of behavioural, cultural, genetic and various other anonymous factors, exploration of which may lead to proper definition of vaginal health and disease.

## Background

Vaginal symptoms, such as discharge, odor and itching are frequently known causes of suffering and discomfiture in reproductive age women. These symptoms can be attributed to recurrent vulvovaginal infections (RVVI), that have not only became an epidemiological and clinical problem, but also include larger social and psychological consequences. The untreated RVVI can lead to complications like infertility, pre-term birth, miscarriages and other infectious diseases [1]. Due to these adverse effects on the reproductive health and well-being of women, vaginal infections have become a major public health concern all over the globe. The emergence and spread of human immunodeficiency virus (HIV) have made the prevention and management of RVVI even more important and urgent. World Health Organization (WHO), IUSTI/WHO and centres for diseases control (CDC) have provided guidelines on vaginal discharge management [2–4]. However, despite of these efforts, the cases of RVVI are still persisting and emerging, probably owing to misdiagnosis and wrong treatment. Literature has suggested fall in *Lactobacilli* predominance and overgrowth of opportunistic pathogens as the reason behind RVVI pathogenesis. *Lactobacilli* have been shown to constitute first line of defence against these pathogens, which by competing with them maintains their low number in vagina, hence suggested to be associated with vaginal health. In spite of this natural primary defence, vaginal infections occur repeatedly. This recommended the need to understand the factors that affect the dynamics and thus composition of microbial communities in vagina in both health and disease conditions, which further will be instrumental for the development of efficient diagnostic and treatment strategies. Over the past few decades, studies have provided some insights into the role of microbial communities inhabiting the vaginal cavity. However, the focus of most of these studies remains the bacterial part of vaginal microbiota both in term of vaginal health and vaginal disease. These studies undervalued the fact that human microbiota also constitutes the fungal part that may equally affect the human health, and other RVVI i.e. vulvovaginal candidiaisis (VVC) and trichomoniasis (TV) are also commonly occurring and clinically important similar to bacterial vaginosis (BV). Thus, through this review, an attempt has been made to analyze vaginal microbiota (VMB) from scratch and to provide an update on its current understanding in relation to health and common RVVI, making the present review first of its kind.

## Selection of literature for review

The potentially relevant studies were retrieved from the Medline/PubMed and Google Scholar. Multiple keywords were used for the literature search both alone as well as in combination. Some of the important keywords used for literature search were microbiota, microbiome, microbial communities, healthy vagina, common vaginal infections, vulvovaginal infections, vaginal ecosystem, vaginal dysbiosis, bacterial vaginosis, vulvovaginal candidiasis, trichomoniasis *et cetera*. Only articles with English language were considered in the present study. The reference lists of retrieved articles were also screened to find relevant articles that were not identified by the initial search strategy.

## Vaginal microbiota in health

### Characteristics and composition of healthy vaginal microbiota

The community of microorganisms that lives interior or on the outer surface of human body forms human microflora/microbiota and their genomic constitution is referred to as human microbiome. The human microbiota usually involves symbionts that are benefitting from the host, but in turn may not affect (commensalism) or may affect positively (mutuality) and negatively (Pathogenic) the functioning, immunity and nourishment of host [5]. Individual microbiome achieved during birth changes throughout life, indicating microbiome specificity. In vagina, the sharp co-operative relationship of microbes with the host provides first line of defence against the migration of opportunistic pathogens. This healthy balance is referred to as eubiosis. However, outweighing of opportunistic pathogens, disrupt this symbiotic balance referred to as dysbiosis that further leads to inflammation. A mutual relationship exists between woman reproductive physiology and vaginal microbiota (VMB) i.e. not only the physiological changes that starts from birth and continues till post-menopause, can affect VMB, but in turn VMB can also affect reproductive physiology [6].

The VMB composition and structure have been described adequately in the literature starting from analyses using light microscopy to high-throughput sequencing techniques [reviewed in 5]. From the very past, the VMB of a healthy reproductive aged women is defined as *Lactobacillus* dominated microflora, producing ample quantity of lactic acid with pH < 4.5 [reviewed in 7]. However, molecular based techniques facilitated the detection of uncultivated and fastidious bacteria that were not previously recognized with conventional techniques, resulting in establishment of unique microbial community state types (CSTs). Based on the abundance and composition of vaginal bacterial species in reproductive age women, these CSTs have been classified into five major types [8, 9]. CST-I, CST-II, CST-III and CST-V are characterised by abundance of *Lactobacillus crispatus, L. gasseri, L. iners* and *L. jensenii* respectively. However, CST-IV is characterised by blend of diverse facultative anaerobes with low levels of *Lactobacilli*. This CST-IV has been further divided into two sub-states CST IV-A and CST IV-B. CST IV-A contains species of genera *Anaerococus, Peptoniphilus, Corynebacterium, Prevotella, Finegoldia* and *Streptococcus*. CST IV-B is characterised by *Atopobium, Gardnerella, Sneathia, Mobiluncus, Megasphera* and other taxa of order *Clostridiales* [8, 9]. Based on Nugent score, CST-IV represents the most common dysbiosis state *i.e.* bacterial vaginosis (discussed later in review). However, this state was also found to be reported in various healthy individuals, predominantly (40%) in black and Hispanic women [8]. Therefore, it is still debateable whether this CST represents a healthy state or an asymptomatic state of BV; further giving emphasis to redefine and distinguish between what is healthy and asymptomatic. Recently, a new genus *Vaginella massiliensis,* a rod-shaped, non-motile; non-sporulous obligate aerobic gram-negative bacteria was cultured from vaginal sample of healthy women [10]. Marseille P2517 is the representative strain of this new genus belonging to the family Flavobacteriaceae and phylum Bacteroidetes.

### Rationales for *Lactobacilli* dominance in healthy VMB

The production of various antimicrobial substances by *Lactobacilli* has been documented as the major rationale behind its predominance in healthy human VMB. These antimicrobial substances include lactic acid, narrow-ranging bacteriocins and wide-ranging hydrogen peroxide (H_2_O_2_). These are suggested to play various important roles in host defence.

#### Lactic acid

*Lactobacilli* produce lactic acid as a result of fermentation of carbohydrates, mainly glycogen, present in the vaginal epithelium of menarchal women. This acidic environment provides protection against infectious diseases by preventing vaginal colonization of potential pathogens. Studies have suggested that *Lactobacilli* abundance acidify the vaginal medium to mean pH, 3.5 ± 0.2, mainly attributed to lactic acid accumulation [11]. The sufficiently protective levels of lactic acid in vagina are mainly dependent on VMB, as host epithelial cells contribute only 4–30% of the whole vaginal lactic acid [12]. Lactic acid is present in vagina in two different isomeric forms i.e. D(–) and L(+). It was suggested that glycogen availability into the vaginal lumen increases due to exfoliation of glycogen-rich epithelial cells by hyaluronidase-1 and matrix metalloproteinase activity [13]. This further causes α-amylase to degrade this available glycogen, that subsequently gets converted into D(–) lactic acid by *Lactobacilli*. However, no correlation of L(+) lactic acid was observed with α-amylase, suggesting that D(–) lactic acid is responsible for maintaining the vaginal pH at ≤ 4.5 and prevents growth of other bacteria. In addition, a recent study indicated that lactic acid, particularly in its L(+) form, suppress HIV-1 infection independent of its pH lowering property [14]. Another study suggested that lactic acid in its both D(–) and L(+) forms induce an anti-inflammatory response of human cervico-vaginal epithelial cells against HIV [15]. Also, both D(–) and L(+) lactic acid were found to inhibit histone deacetylases, thereby increasing DNA repair by regulating transcription of genes associated with it [16].

In addition, different studies have suggested the role of lactic acid in inhibiting wide variety of infections including *Chlamydia trachomatis* infection, Herpes simplex virus type 2 (HSV-2), HIV, HIV-1 and broad range of BV-associated microbes [14, 17–19]. Lactic acid have been shown to affect host immune responses through different mechanisms including eliciting significant increases in the production of the anti-inflammatory mediator i.e. interleukin-1 receptor antagonist (IL-1RA) from vaginal epithelial cells, inhibiting production of pro-inflammatory mediators i.e. IL-6, IL-8, tumor necrosis factor alpha (TNFα), RANTES (regulated on activation, normal T cell expressed and secreted) and macrophage inflammatory protein-3 alpha (MIP3α) [15], releasing transforming growth factor beta (TGF-β) to stimulate antiviral response [20], stimulating the T helper 17 (Th17) T lymphocyte pathway via IL-23 production on exposure to bacterial lipopolysaccharide [21] and building up cytosolic lactic acid that blocks the production of cyclic adenosine monophosphate (cAMP) leading to increased autophagy in epithelial cells for the degradation of intracellular microbes and upholding homeostasis [22]. Overall, these studies suggested different defensive properties of lactic acid that further depends on its isomeric state. These properties either independently or together determine the host susceptibility and consequently host–microbiota relationship.

#### Bacteriocins

Bacteriocins including Bacteriocins IIa, IIc, J46, acidocin lF221A, gassericin T and type-A lantibiotic are proteinaceous substances with bactericidal activity, synthesized by *Lactobacilli* particularly *L. crispatus* and *L. gasseri* [23]. Bacteriocins permeablize the cell membrane of non-indigenous pathogenic organisms i.e. *S. aureus, Klebsiella spp., E. faecalis, E. coli* and plays a major role in preventing their growth [23].

#### Hydrogen peroxide (H_2_O_2_)

H_2_O_2_ is another antimicrobial substance that is known to be produced in vitro by many *Lactobacillus* species in the presence of oxygen (O_2_) [24]. However, the levels of dissolved O_2_ are low in vagina maintaining an anaerobic atmosphere. So, the accumulation of H_2_O_2_ to a level sufficient to provide toxicity to pathogens is doubtful in vagina. O’Hanlon et al. [17] further complemented this presumption and showed that H_2_O_2_ production is not a considerable factor in preventing pathogens. The study demonstrated that under low levels of O_2_ i.e. anaerobic conditions of vagina, H_2_O_2_ had no noticeable effect on bacteria associated with BV [17]. Additionally, high levels of H_2_O_2_ were found to be more harmful to *Lactobacilli* responsible for eubiosis than bacteria responsible for dysbiosis [17]. In contrast to this study, some earlier studies have shown that H_2_O_2_ producing vaginal *Lactobacilli* are more likely to be protective against BV and suppressing *Candida* overgrowth and invasive hyphal formation [25]. Overall, suggesting that lactic acid and bacteriocins production mainly contributes to the protective role of *Lactobacilli* in VMB. However, the role of H_2_O_2_ in VMB protection is still debatable signifying its role as a substitute marker for other, yet unknown, physiological factors.

A recent study has suggested the mechanisms by which *Lactobacillus* suppress HIV type-1 (HIV-1) infection [14]. The acidification provided by *Lactobacilli* abundance was found to be efficiently mimicked by culture medium with Hydrochloric acid (HCl) of same mean pH (between 3.8 and 4.6) in preventing HIV-1 replication. However, the pH of both medium i.e. *Lactobacillus* conditioned medium and culture medium with HCl was diluted fivefold to reach pH 6.9, the effect was not mimicked by diluted HCl medium. As fivefold diluted *Lactobacillus* medium was still suppressive for HIV-1 infection, acting as a sink diminishing the amount of free virions, while diluted HCl medium was not. This suggested the possibility of other multiple factors and mechanisms responsible for the virucidal effect of *Lactobacilli* and its predominance in VMB that are yet to be discovered.

### *Candida* live as commensal in healthy vagina

The VMB of healthy reproductive aged women also constitute fungal part designated as the “vaginal mycobiota” and its genomic constitution as “vaginal mycobiome” [26]. Researchers using culture-dependent older techniques recovered vaginal fungi in ≈ 20% of the asymptomatic women. Certainly, the predominant part of this mycobiota was occupied by *C. albicans* (72–91%) followed by non-*albicans Candida* (NAC) species including *C. glabrata, C. tropicalis and C. parapsilosis* [27]. However, the complexity of vaginal mycobiota remains unrecognized due to the limitations of conventional approaches. Only few studies regarding the composition and diversity of vaginal mycobiome were based on high-throughput sequencing techniques. In fact, the first study based on pyrosequencing revealing the vaginal myco-communities in asymptomatic women of Estonia came in print in 2013 [26]. The study recovered *Candida* species in 64.5% of the participants, which is significantly higher than the frequency (20%) found in asymptomatic healthy women, investigated with earlier culture based techniques [26, 27]. Also in full agreement with aforementioned earlier studies, the predominant part of this mycobiota, as predicted, was *C. albicans* (82%). Moreover, the NAC species detected in this study were *C. dubliniensis, C. parapsilosis, C. krusei* and *Candida* sp. VI04616. Other than *Candida* species, this study also found 38% of undesignated operational taxonomic units for which no taxonomic assignment were available, emphasizing to a serious dilemma of molecular based fungal taxonomy. Though recent studies have uncovered the underestimated diversity of vaginal mycobiota, *C. albicans* again remain the ultimate performer, further proposing future follow up studies [26, 28].

*Candida* species, though present in healthy women, still designated as opportunistic pathogen, due to their high prevalence (85–95%) in patients suffering from VVC, which is the second most prevalent dysbiosis after BV [1]. Thus, it remains to be determined whether the presence of *C. species* conveys a benefit to the host by maintaining balanced microbiota (eubiosis) or is responsible for causing VVC (dysbiosis). The answer to this may lie in the unique characteristic of *Candida* i.e. dimorphic transition. This feature allows *Candida* to undergo a morphological change from a round-ovoid yeast cell to a hyphal mycelial growing organism, further permitting it to live a dual lifestyle both as commensal as well as pathogen. The yeast form is usually found in healthy asymptomatic women in contrast to hyphal form, which has consistently been isolated from cases of severe VVC. This further supports the association of yeast form with commensalism and hyphal form with pathogenicity [29]. There are many factors that are responsible for *Candida* commensalism and one main factor is nutrition. Different studies have indicated the effect of carbon source on cell wall structure, which further affect the virulence of *Candida* and its interaction with host immune cells [30, 31]. *Candida* can utilize different carbon sources including glycogen, its breakdown products and even lactic acid as an alternative carbon source, which makes *Candida* highly flexible to dietary shifts in vagina [32]. Recently, a study suggested that growing *Candida* on lactate as its only nutrient source lead to modulation of its interactions with immune cells dampens phagocytosis, mount IL-10 and decline IL-17 production making it less vulnerable to immune responses [33]. Other studies have confirmed these findings, recommending that as *Candida* grows, it reduces the immune responses generated against it and thus endorse its own existence, establishing commensalism [34, 35].

### *Candida*-bacteria interactions

*Candida* and bacteria plays a major role in sustaining *Candida* commensal form through five main clinically important bacteria-fungal interactions that commonly occur in human microbiota as reviewed by Peleg et al. [36]. These interactions also take place in VMB and include physical interactions, exchanges of chemicals, use of metabolic by-products, milieu variations and modification of the host immune response [25, 37, 38]. Studies based on co-culturing of vaginal yeast and bacteria suggested that bacteria inhibit *Candida* yeast-to-hyphae switch, maintaining its low number in VMB and compete with *Candida* cells for adhesion sites on epithelial receptors owing to its higher affinity [25, 37]. Also, Low pH and bactericidal compounds secreted by *Lactobacilli* tends to suppress *Candida* overgrowth and its transition from avirulent yeast form to virulent hyphal form [25, 39]. Recently, a study showed that *L. crispatus,* one of the dominant member of VMB, can diminish *C. albicans* virulence and enhance the local immune response of vaginal epithelial cells by modulating immune cytokines and chemokines profile i.e. upregulating IL-2, IL-6, IL-17 and downregulating IL-8 [38]. Overall, these studies suggested that bacteriobiota of VMB arbitrate tolerance to *C. albicans* in vagina. To complement this, different studies have linked the scarcity of *Lactobacilli* with susceptibility to symptomatic VVC and other lower genital tract infections including BV and HIV [40, 41]. All these findings suggests that *Lactobacillus* abundance and low *Candida* number along with their interactions play an important role in maintaining microbiota balance and disturbance in this may lead to vaginal infections.

### Vaginal mucosa

The vaginal mucosa as a caretaker protects the lower genital tract from harmful pathogens including HIV [42]. Surfactant protein A (SP-A), produced in vaginal mucosa provides host defence by opsonizing pathogens, altering levels of pro-inflammatory cytokines, stimulating oxidative burst by phagocytic cells and promoting differentiation of antigen presenting cells, there by linking innate with adaptive immunity [43]. In addition, studies have shown that VMB composition also determines vaginal mucosa defence property which is shown to be strengthening by *L. crispatus* dominated VMB and weaken by *L. iners* dominated VMB [42, 44]. Other than this, most of the *Candida*-bacteria interactions likely to take place in vaginal mucosa where *C. albicans* property of biofilm formation was shown to be inhibited by *Lactobacilli* [45] though, only recently no in vivo biofilm formation by vaginal *Candida* species has been claimed based on histopathogical evidences [46]. Thus, change in quality and quantity of standard SP-A and vaginal microbial community composition can affect the gate keeping property of vaginal mucosa and therefore susceptibility to vaginal infections.

### VMB throughout the women’s life

**Fig. 1:**
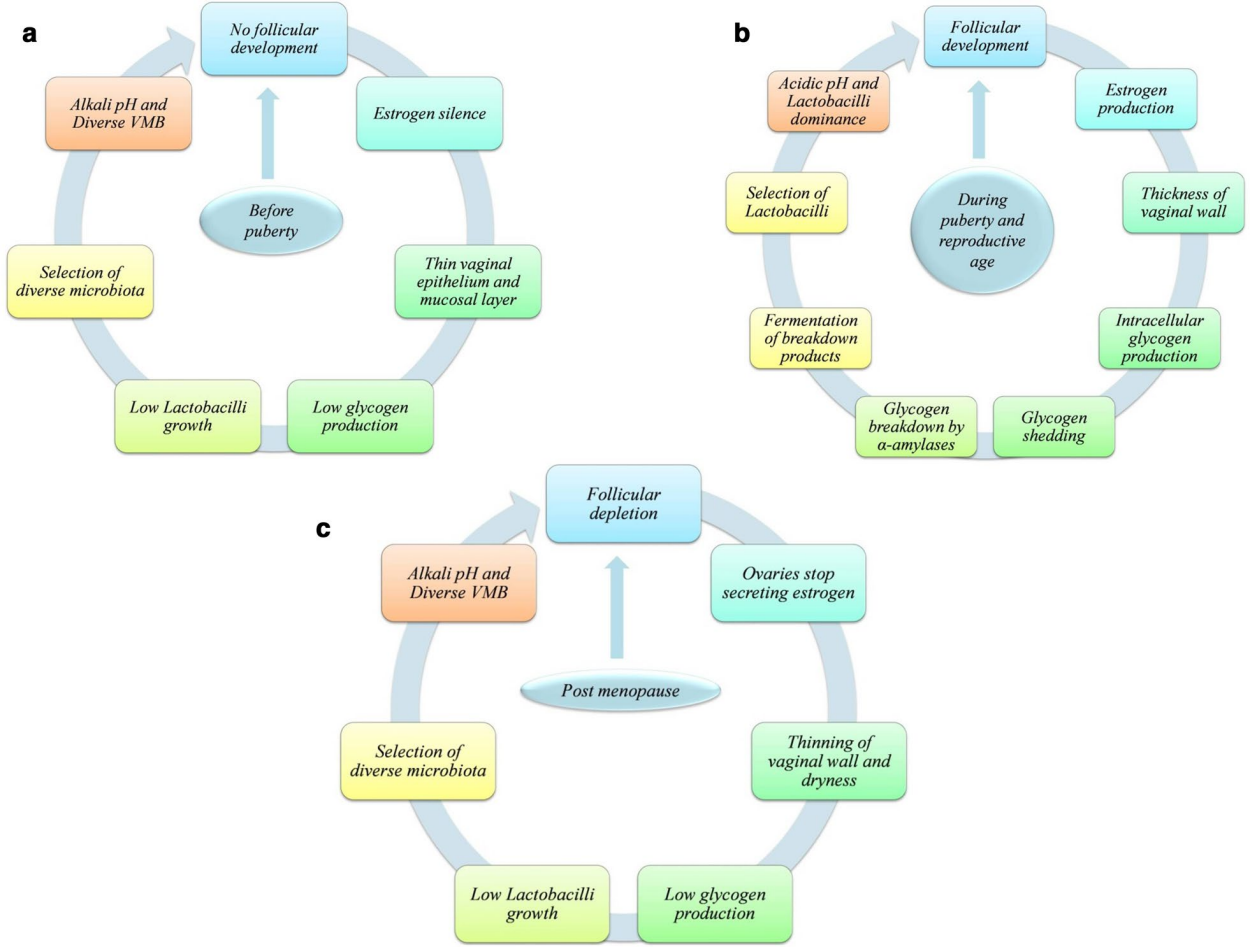
Host physiology effects VMB throughout a women’s life i.e. **a** Before puberty, **b** during puberty and reproductive age and **c** post-menopause

As women ages, its VMB composition changes significantly together with vaginal epithelium structure, and hormones have been suggested as main players (Fig. 1) [6, 9, 47].

#### At birth

The initial colonization of microbes takes place from mother vagina, if delivered normally, or from the hands of person that first hold the infant after caesarean [48]. Though little information is available, it is suggested that within weeks these microbes differentiate into different communities and establish in skin, gut and vagina [48]. There is evidence that infants have residual amount of circulating estrogen of mother which may be consumed by some *Lactobacillus* species for metabolism [49].

#### Before puberty

The vagina of child has CST-IV of VMB, neutral pH and thin stratified squamous epithelium roofed with thin mucosal layer [50]. Till 10 years of age, ovaries do not produce hormones (estrogen silence), resulting in low glycogen levels, consequently low growth of *Lactobacilli* and thus alkaline vaginal milieu. However, after 13 years of age, adrenal and gonadal maturation leads to pubertal changes in the vulva and vagina.

#### During puberty and reproductive age

Follicular development in puberty results in estrogen production. High estrogen promotes the thickness of vaginal epithelium along with mucosal layer and intracellular glycogen production [6]. Shedding of the glycogen rich epithelial cells in response to reproductive hormones leads to availability of free glycogen, which is further processed by α-amylases [32]. Available glycogen and its breakdown products i.e. maltose and maltotriose causes the selection of *Lactobacilli* capable of fermenting glycogen to lactic acid resulting in vaginal acidification and *Lactobacilli* dominance in VMB (CST I, II, II and V), with exception of CST IV microbial diversity found in some healthy women [6, 11]. Both the culture dependent and independent studies tried to uncover the complex and dynamic nature of vgainal microbial communities. The culture dependent studies have shown day to day variations in VMB composition [51, 52]. These findings were replicated by culture-independent studies based on longitudinal analyses of women who have done self sampling for weeks [9, 47, 53]. These studies have shown that some women showed switching between different CSTs over a short span, while others remain relatively consistent particularly the CST dominated by *L. crispatus* [9, 53]. These CST conversions were shown to be eliciting by menstruation [9, 47]. In 81% of menstrual cycles, the levels of *G. vaginalis* along with *L. iners* was shown to boost significantly with menses and decreases toward its end, while vice versa occurs with levels of the other two *Lactobacilli* namely *L. crispatus* and *L. jensenii* [47]. This can be attributed to the availability of iron during menses that improves the growth of *G. vaginalis* and *L. iners,* as easy culturing of both have been observed in blood agar medium [54]. Throughout a woman’s reproductive age, glycogen levels remain high, which steadily turn down when reaching menopause [55].

#### Post-menopause

Post-menopause leads to number of changes in the vaginal milieu that includes lowering of estrogen and glycogen levels, thinning of vaginal epithelium as that of pre-puberty stage, VMB shifting from *Lactobacilli* to microbial diversity (CST-IV), rise in pH, reduced vaginal secretions, dryness and dyspareunia [56]. Thus, VMB of girls at pre-puberty stage resembles with VMB of menopausal and post-menopausal women suggesting that reproductive physiology plays a major role in determination of women VMB [57]. However, vaginal milieu in postmenopausal women was found to be restored by application of estrogen cream, similar to that of reproductive age women [58].

Overall, the work highlighted the vibrant nature of VMB. However, the women considered in these studies were healthy indicating that CST’s compositions are not the sole indicator of dysbiosis, rather there are some uncharacterized factors that are needed to be resolve immedicately [9].

## Vaginal microbiota in recurrent vulvovaginal infections (RVVI)

Diversity in VMB causes dysbiosis, i.e. different communities of VMB compete with each other leading to the selection of community capable of tolerating adverse conditions. This process may lead to damage or fall in number of beneficial *Lactobacillus* species, thus increasing host susceptibility to opportunistic pathogens either normally present in human VMB in lower quantity or are sexually transmitted. As this nasty cycle remains unchecked for long period, it may magnify the effect of dysbiosis leading to persistent imbalance with chronic inflammation i.e. vaginitis or vulvovaginal infections (VVI). These VVI are usually named on the predominant community or infectious agent present in vagina responsible for causing severe symptoms, like excessive vaginal discharge (offensive/non-offensive), vulval itching, fissuring, soreness, erythema, oedema, dysuria, dyspareunia and skin lesions. Of all these symptoms, an abnormal vaginal discharge is the key trait of these infections. Bacterial Vaginosis (BV), Vulvovaginal Candidiasis (VVC) and Trichomoniasis (TV) are the three main commonly prevalent VVI [59–62] and the centre point of this review. Moreover, recurrent VVI (RVVI) i.e. repeated experiences of common vaginal infections is emerging and is the major concern for researchers these days. For example, the repetition rates as high as 30–50% within 3 months is designated as recurrent BV (RBV) while ≥ 4 repetitive episodes of VVC in 12-months are referred as recurrent VVC (RVVC) [1, 63]. Similarly, cases of recurring TV (RTV) have also been reported with recurrence rates as high as 5–8% within 2 months of initial diagnosis [64].

### Bacterial vaginosis (BV)

#### VMB composition in BV

**Fig. 2:**
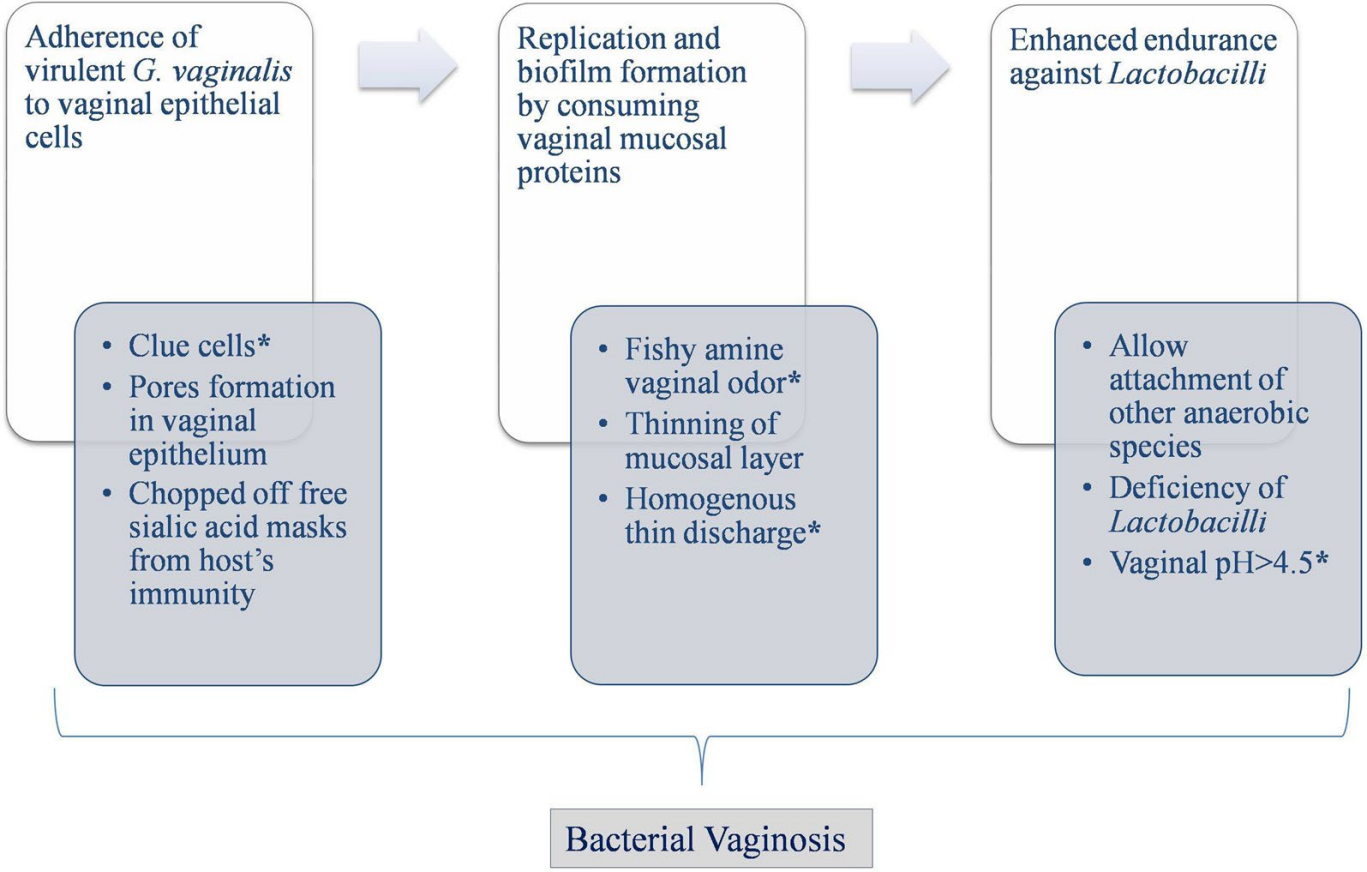
Pathway implicated in the pathogenesis of bacterial vaginosis, ‘*’ indicates four clinical symptoms of BV described by Amsel’s criteria

As aforementioned, BV is a dysbiotic state characterised by deficiency of lactic acid producing bacteria and sufficiency of anaerobic bacterial diversity. This anaerobic diversity includes species of *Anaerococcus*, *Atopobium*, *Bacteroides*, Bacterial vaginosis-associated bacteria type 1 (BVAB1), BVAB2, BVAB3, *Gardnerella*, *Leptotrichia*, *Mobiluncus, Mycoplasma, Mobiluncus, Peptostreptococcus, Peptoniphilus, Prevotella* and *Sneathia*. Advancement in molecular based technology has been continually expanding the list of potential agents associated with BV, including the non-cultivable bacteriobiota that has limited the use of earlier culture based techniques [65]. For example, very recently three strains namely “*Olegusella massiliensis*” strain KHD7^T^, “*Ezakiella massiliensis*” strain Marseille P2951^T^ and “*Corynebacterium fournierii*” strain Marseille P2948^T^ were isolated from the VMB of woman with BV [66–68]. All these three strains were gram-positive with strain KHD7^T^ and strain Marseille P2951^T^ being strictly anaerobic, while strain Marseille P2948^T^ was a facultative anaerobe, that grows very well under aerobic conditions and feeble under anaerobic conditions. Furthermore, scarcity of both isomeric forms of lactic acid were found in women diagnosed with BV, resulting in vaginal pH > 4.5 [69]. The latter is one of the four clinical symptoms of BV described by Amsel’s criteria for clinical diagnosis of BV (Fig. 2) [70].

#### VMB’s energy source and *G. vaginalis* as a key component

In addition, metabolites assessment has suggested the anaerobic diversity in BV patients was found to be dependent on nitrogen source rather than on carbon source for their energy requirement. Catabolism of amino acids results in amines that is responsible for fishy amine vaginal odor observed in BV women, which is one of the four symptoms of BV described by Amsel’s criteria [71]. Not only this, catabolism of mucosal proteins by bacterial enzymes leads to the thinning of mucosal layer, making it less defensive and results in production of thin homogenous discharge lining the vaginal walls, another symptom of BV described by Amsel’s criteria [71]. Recently, *G. vaginalis* was suggested to be the key component of anaerobic diversity responsible for symptoms found in BV patients [72, 73]. *G. vaginalis* adhere to the surface of vaginal epithelial cells leading to the formation of clue cells, the fourth symptom of BV described by Amsel’s criteria [74]. Afterwards, *G. vaginalis* starts replicating in vaginal mucosa by consuming it and developing a thin biofilm that allow the attachment of other species [75]. This biofilm formation enhances the endurance of *G. vaginalis* against beneficial bacteria of VMB and antibacterial regimens [76].

#### Virulent forms and factors of *G.* *vaginalis*

As aforesaid, *G*. *vaginalis* is also a part of CST-IV of healthy VMB that challenges it as a cause for BV. This inconsistency led the researchers hypothesised that *G*. *vaginalis* might exists in different virulent forms and found that genotypic and phenotypic diversity actually exist in *G. vaginalis* [77, 78]. This has been further affirmed by a recent study, proposing the existence of ten strains of *G. vaginalis* (β-galactosidase positive) [79]. Apart from this, *G. vaginalis* strains expressing sialidase A gene has shown to be associated with BV and biofilm formation [73]. Sialidase A gene codes for enzyme sialidases that chop off sialic acid from glycoproteins of vaginal mucosal layer leaving behind basic glycan structure that serves as adherent for *G. vaginalis* [80]. Additionally, the free sialic acid residues provide nutrition to the bacteria as well as incorporate in surface, masking it from host’s immune responses [81]. Adherence, nutrition and masking allow proliferation of *G. vaginalis* resulting in continuation of this process subsequently leading to degradation and thinning of protective mucosal barrier of vagina [80]. Besides this, *G. vaginalis* also produces vaginolysin and prolidase, other characterized virulence factors of this bacterium. Vaginolysin is a cholesterol-binding cytolysin, primarily known as hemolysin, which encourages cell lysis through a colloid osmotic mechanism that leads to pores formation in the vaginal epithelium, contributing to BV signs and symptoms including vaginal fissures or sores [82]. Another virulence factor, prolidase, is a dipeptidase that degrades proline specific dipeptides contributing in amine odor observed in BV as mentioned above [83]. Apart from this, a new mucolytic enzyme, i.e. glycosulfatase, that chop off sulphate from glycosulphates, was found to be associated with BV [84]. The theory behind the removal of targeted residues is same in both glycosulfatases and sialidases.

#### G. vaginalis is not sole responsible for BV

Moreover other bacteria, including *Bacteroides* and *Prevotella* species, from anaerobic diversity of BV also produces sialidases and glycosulphates [85]. In addition, recently a culture-independent study based on longitudinal analyses has linked increased vaginal bacterial diversity in BV to vaginal inflammation [86]. The study described the longitudinal changes in VMB and immune mediators of African women with and without BV for 8 weeks and found that women with BV had significantly high concentrations of bacterial diversity and of pro-inflammatory interleukins including IL-1β and IL-12 (p70), that can cause inflammation and thus health damage. Also, a recent study has shown the occurrence of localised inflammation due to RBV [87]. Overall, these studies suggest that *G. vaginalis* is not solely responsible for BV. In fact, BV is a multimicrobial disease caused by a variety of communities formed from diverse microbes that synergistically lead to same physiological symptoms. As per the definition of syndrome, all these symptoms together leads to clinical diagnosis of BV [Amsel’s criteria, 70]. However, one statement i.e. same microbial diversity also exists in healthy/asymptomatic women (i.e. CST-IV of VMB) makes all the important findings looks vague. No definite conclusion could be drawn regarding anaerobic diversity acting as pathogen or opportunistic organisms, suggesting the presence of additional factors that are yet to be determined.

#### Another bewildered bacterial dysbiosis

Additionally, another dysbiotic condition known as aerobic vaginitis (AV) is repeatedly baffled with BV. This is because, both resembles with CST-IV of VMB i.e. lacking significant number of *Lactobacilli* and hence pH > 4.5. However, literature differ these two conditions based on the type of microbial diversity and inflammation. While, BV is designated by the presence of strictly anaerobic bacteria and as a non-inflammatory condition, AV is designated by the presence of aerobic intestinal bacteria including *Escherichia coli*, *Staphylococcus aureus*, group B *Streptococcus* (*Streptococcus agalactiae*) or *Enterococcus* and as inflammatory condition [88]. Infact, a severe AV is the same as desquamative inflammatory vaginitis (DIV), which is an unprecedented serious type of chronic purulent vaginitis of unknown aetiology causing non-specific signs and symptoms with abnormal vaginal flora, elevated pH and increased inflammation [89]. Although, nearly always high numbers of *Streptococcus agalactiae* and others species are found, AV and DIV are probably immunological disorders of the vagina and not infections owing to their positive response to anti-inflammtory agents. Apart from these, Group A streptococcal vaginitis is a recognized cause of vaginitis among prepubertal girls and an emerging disease entity in adult women as well, therefore should be considered a diagnosis when more common causes of vaginitis have been precluded [reviewed in 90].

### Vulvovaginal candidiasis (VVC)

#### Debabtable VMB composition in VVC

Literature has reported normal vaginal pH (pH ~ 4.5) during VVC, indicating the presence of sufficient number of *Lactobacilli* to maintain the acidification of vagina [3]. In accordance to this observation, a study has reported elevated L(+) lactic acid levels in women with VVC [69]. Studies by different groups found no difference in VMB of women with and without VVC and were found to be dominated by *Lactobacilli* [91, 92]. A recent study by Wu et al. [93] found *Lactobacilli* dominant VMB in patients with severe VVC. All these findings support the presence of *Lactobacilli* dominance in women with VVC, suggesting their futile role in providing defence against VVC. Indeed, a study suggested that the probability of getting VVC increases 4 times with *Lactobacilli* colonization [94]. However, in contrast to these findings, a study found low density of *Lactobacilli* in RVVC women relative to controls [95]. This inconsistency in results provided by culture based studies was confirmed by the only study based on next generation sequencing. This study revealed diverse microbial community patterns in VMB of women with VVC and found that VMB of VVC did not show any typical pattern as found in VMB of healthy women and of BV [96]. In fact, it ranges from a CST dominated with *Lactobacillus* (healthy women) to CST with anaerobic diversity (BV). Thus, the part of VMB in VVC is debatable depicting its complex nature in VVC.

#### Co-ordination of stimuli for VVC pathogenesis

**Fig. 3:**
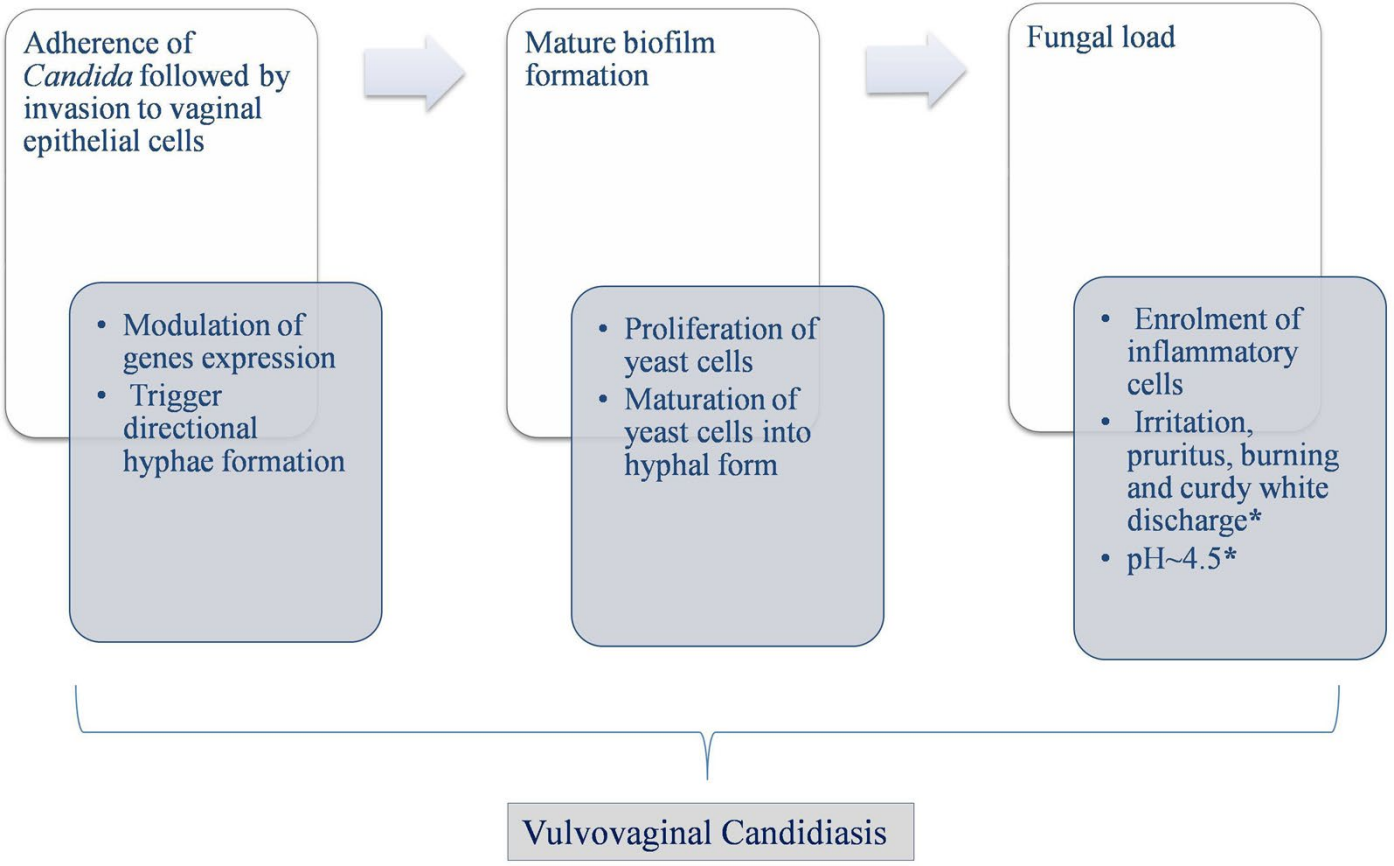
Pathway implicated in the pathogenesis of vulvovaginal candidiasis, ‘*’ indicates clinical symptoms of VVC

VVC is dysbiotic state typified by the excessive growth of *Candida* species, whose morphogenetic transitions to mycelial form is an opening step in pathogenesis [97]. Just like in BV, pathogenesis of VVC involves three steps i.e. adherence followed by invasion to epithelial cells, biofilm formation and secretion of virulence factors (Fig. 3) [29]. Studies have shown co-ordination of stimuli i.e. starvation, pH ≥ neutrality, 37 °C temperature and low *Candida* cell densities (< 10^7^cells/mL) in modulating *Candida* gene expression profile to trigger hyphae formation, followed by adherence to vaginal epithelial cells and further pathogenesis [39, 98]. These genes encode for the proteins including agglutinin-like sequence protein (AlS3), secreted aspartic proteases (SAP 4 to 6), hyphal wall protein (Hwp1) and the hypha associated proteins (HGC1, Ece1 and Hyr1) [39, 99]. Out of these, two proteins i.e. AlS3 and Hwp1 act as ‘adhesins’, facilitating the adherence of *C. albicans* to host epithelial cells [100]. Following adhesion, *Candida* invades the host cells with the help of two proteins namely AlS3 and Ssa1, acting as ‘invasins’ [101, 102].

#### Effect of nutrient starvation and surrounding pH on Candida

Phagocytes create an environment of nutrient starvation for *Candida* [103]. In response of which *Candida* activate glyoxylate pathways for the synthesis of carbohydrates that plays a role in gluconeogensis [104]. Inspite of this, if *C. albicans* sense lack of carbohydrates, it starts using alternative metabolic pathways to consume proteins and lipids. Consumption of proteins liberates ammonia, resulting in increased pH, that auto-induce hyphae formation [92]. This mechanism not only allows *Candida* to grow under starvation but also cause hyphae formation, external pH modulation and thus increasing its virulence [31]. This property allows *Candida* to survive and escape phagocytes by causing host cell death by two different mechanisms including pyroptosis and membrane rupturing of phagocytes [105]. *C. albicans* can not only modulate the external pH, indeed, it can also sense and adapt to surrounding pH with the help of its pH sensitive proteins including Phr1 and Phr2 [106]. However, these proteins along with nutrient gaining property of *Candida* yeast get impaired under stress provided by neutral to alkaline pH [106]. This shows the effect of surrounding pH on *Candida* morphology with acidic pH maintaining yeast cell growth and neutral to alkaline pH leading to hyphal growth [106].

#### Contact sensing and biofilm formation

Additionally, contact sensing is also considered as crucial factor in triggering thigmotropism, i.e. directional hyphae growth and mature biofilm formation [107, 108]. A study has shown importance of directional growth of *C. albicans* for normal virulence, active penetration and complete damage of epithelial cells [109]. Just like BV associated bacteria, *Candida* is competent in making mature biofilms on mucosal epithelial surface after adherence [110, 111]. It involves a chronological process of yeast cells proliferation, followed by maturation of upper yeast cells into hyphal form and subsequently building of surface milieu [108]. This biofilm formation is under the control of certain transcription regulators namely Efg1, Tec1, Bcr1, Rob1, Ndt80 and Brg1 [108, 112]. Eliminating any of these factors may lead to faulty biofilm formation in vivo [113]. Additionally, AlS3 and Hwp1 also play a complementary role in biofilm formation [112]. This illustrates the underlying role of AlS3 in *Candida* pathogenesis performing three roles as adhesins, invasins and in biofilm formation. Above a certain threshold of fungal load, enrolment of inflammatory cells starts, that consequently leads to vaginal symptoms including irritation, pruritus, burning and curdy white discharge [114]. The later involves the detachment of hyphae along with recruited inflammatory cells, lysed cells debris and vaginal liquid making the curdy white vaginal discharge, which is one of the characteristic clinical symptoms of VVC.

#### Virulence factors and contradiction in view of Candida’s biofilm formation

The biofilm formation by *Candida* is a big issue, as it provides heightened virulence and resistance against the host immune responses, antifungal agents, consequently leading to recurrent infections and antifungal treatment failures [115]. Metabolic flexibility, compound biofilm structure, surface milieu of biofilm is some of the factors responsible for biofilm resistance, while diffusion of yeast cells from biofilm contributes to virulence [116]. Though, *Candida*-biofilms have been shown in a myriad of experiments in vitro as well as on catheters and prosthetic surfaces in vivo, no proof of in situ, in vivo *Candida* biofilm presence has been reported on the vaginal surface, regardless of the likelihood that *Candida* biofilms are a crucial factor in VVC pathogenesis. Paradoxically, a recent study contradicts this well-known supposition, suggesting no resemblance of in vivo histopathological lesions in vagina with biofilms in VVC [46]. This is the reason an alternate understanding what a *Candida*-biofilm is required. The other known virulence factors include secreted hydrolases namely protease and phospholipase that facilitate invasion and nutrients acquisition [117, 118]. The well-studied proteolytic enzymes employed by *Candida* include family of SAPs that is known to cause epithelial cell damage and systemic infections [118]. Recently, a new cytolytic peptide toxin namely Candidalysin has been proposed to be secreted by *C. albicans,* that leads to epithelial cell damage and triggers host immune responses [119]. The strains lacking this toxin were found to be avirulent, suggesting it as a causal determinant of virulent strains. Overall, researchers are still not able to fully uncover the mechanisms behind *Candida* pathogenesis leading to VVC. The factors responsible for pathogenic morphology transitions are still difficult to understand and the system behind immune-protection are still debatable.

#### Genetic variability in Candida species

However, efforts are being made for better understanding of these factors based on comparative genomics. As, there are strains of *Candida* that are avirulent and do not have the capacity to undergo dimorphic transition [120]. Earlier study based on longitudinal karyotyping suggested the persistent presence of single *Candida* strain in VMB, experiencing genetic microevolution and was responsible for repeated episodes of VVC [121]. This fungal persistence and resistance was found to be associated with variety of genetic variations found in *Candida* species including Single nucleotide polymorphisms (SNPs), reshuffling, heterozygosity loss and copy number variations (CNVs) [122]. There are two studies that are based on whole genome sequence analysis of *Candida* species isolated from women with VVC [123, 124]. These studies recommended the modulation of genetic system under the influence of unique host environment that affects the growth of *Candida* species. Without a doubt, these comparative genomics analyses of *Candida* will reveal the various anonymous factors and genomic variations responsible for dimorphic transitions that occur in response to the host dynamic vaginal milieu. However, no VMB differences were found in women with and without VVC suggests the exploration of host itself that will improve our understanding behind the pathogenesis of VVC.

### Trichomoniasis/Trichomonas vaginitis (TV)

#### Initiation of pathogenesis by parasite

**Fig. 4:**
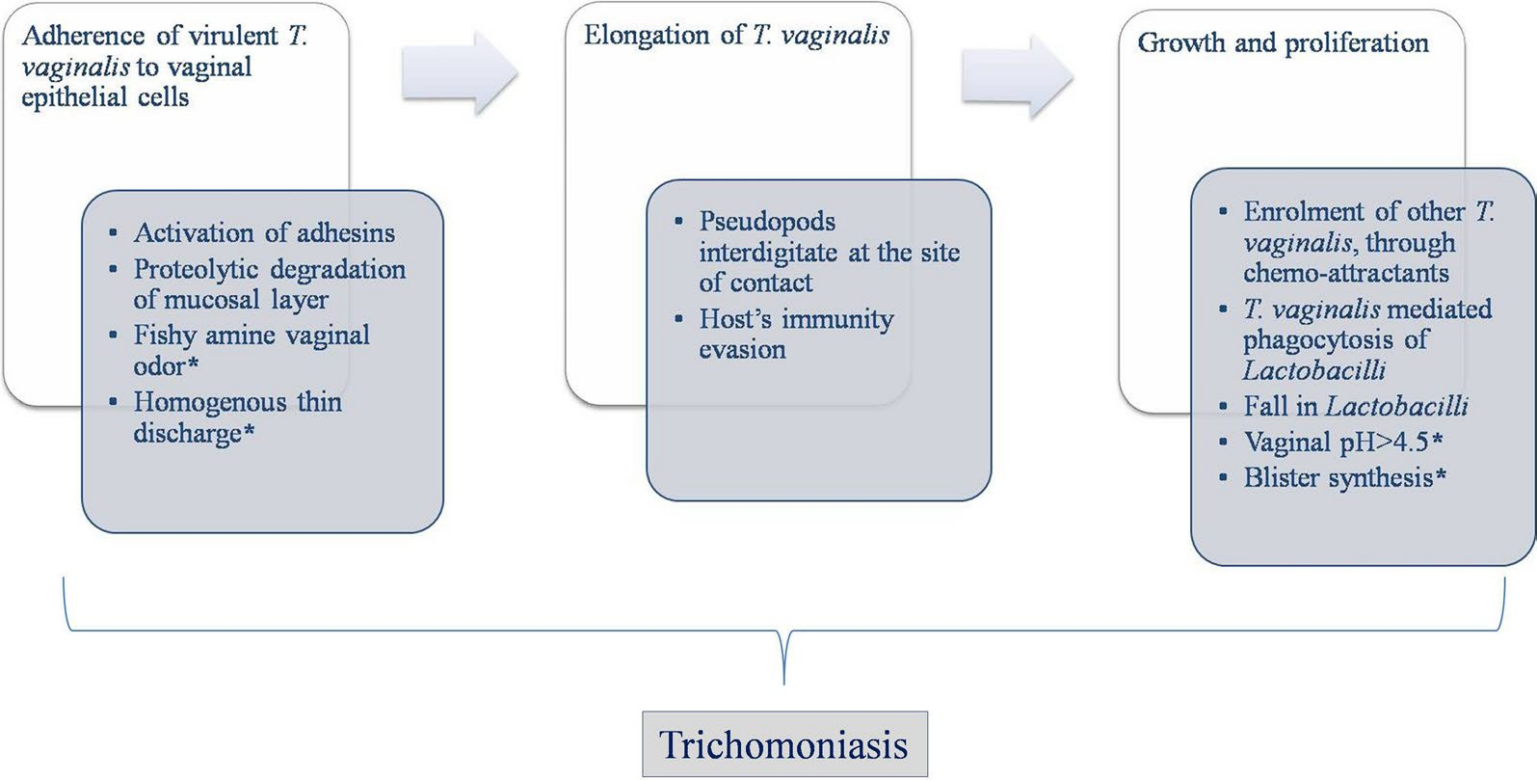
Pathway implicated in the pathogenesis of trichomoniasis, ‘*’ indicates clinical symptoms of TV

Another common type of RVVI is Trichomoniasis. As the name suggest, it is caused by *Trichomonas vaginalis,* the prevalent pathogen present in vagina during infection. *T. vaginalis* is an anaerobic flagellated parasite which normally exists as trophozoite but sporadically in amoeboid form. *T. vaginalis* pass on sexually amongst human, its only reported host [125, 126]. The first host surface that came across by trichomonads is the host vaginal mucosal layer. Network of Mucin, a key protein component of mucosal layer, serve as a gate keeper to pathogen invasion. However, this barrier was broken down by proteolytic degradation by mucinases (cysteine-like peptidases) found in *T. vaginalis* [127]. This further permits the contact of *T. vaginalis* to the underlying vaginal epithelium and its components including phosphoglucomutase, enolase, lamimin binding, fibronectin and α-actinin that equivalently plays a role in its cytoadherence [128]. Just like first two dysbiotic states, the initial step in pathogenesis of trichomoniasis is adhesion to vaginal epithelial cells via opposite site of undulating membrane (Fig. 4) [129]. A lipophosphoglycan (LPG) dominated dense glycocalyx is anchored to the plasma membrane of *T. vaginalis* via inositol phosphoceramide [130]. This LPG supports *T. vaginalis* adherence to the vaginal epithelium [131]. The process of adhesion involves two kinds of proteins namely adhesins and cysteine proteases. The former includes five types namely AP23, AP33, AP51, AP65 and AP120, while cysteine proteases involve 11 to 23 different proteins [132, 133]. Adhesins are usually present on the surface of trichomonds in inactive form [134]. The activation process requires unmasking of adhesins from the specific proteins covering it, with the help of cysteine proteases [126]. The activated adhesins interact with vaginal epithelial cells and mediate attachment of trichomonads with it [134]. This binding leads to the elongation of *T. vaginalis* making it amoeboid with the formation of pseudopods that interdigitate with target cells at the site of contact [135]. Attachment of one trichomonad is shown to facilitate the enrolment of others, through the secretion of chemo-attractant [136]. A recent study reported the release of membrane vesicles from *T. vaginalis* surface that interact with target host cells through receptor or through fusion with target cell membrane to deliver certain signals important for this parasite pathogenesis [137].

#### Important nutrients as a regulatory source of virulence

Just like other microbes, the *T. vaginalis* use carbohydrates as its favoured carbon source for growth and nutrition, but in the absence of sugars, its switches to lipids and amino acid metabolism [138]. However, *T. vaginalis* do not have the ability to synthesise lipids, they mediate lysis of host erythrocytes (similar to the hameolysis of BV), which is shown to be maximum at normal vaginal pH of 4.5 and use it as a source of fatty acids [139]. Moreover, *T. vaginalis* also acquire CD59 from erythrocytes; thereby evading itself from host complement mediated killing [140]. This hameolysis is facilitated by the cysteine proteases; another virulent property of *T. vaginalis*, which also helps in iron acquisition by *T. vaginalis* [141, 142]. Iron is considered to be an important nutrient of *T. vaginalis* that upregulates the levels of various adhesins and proteases, thereby amplifying the loads of infection [141]. Recently, a 120 kDa protein named pyruvate ferredoxin oxidoreductase (PFOR) has been shown to influence pathogenicity of *T. vaginalis* by modulating adhesion, proliferation and blister synthesis [143]. Moreover, this PFOR has been verified to show structural and functional homology with AP120, a novel adhesin of *T. vaginalis* induced by iron [133]. Other than this, adhesins including AP51 and AP65 of *T. vaginalis* were also shown to serve as heme and haemoglobin binding proteins, validating their multifunctional role in *T. vaginalis* [144]. A part from this, iron up-regulated cysteine proteases help in evasion of host’s immune responses by degrading its various components that includes, subclasses of host antibodies (IgG and IgA), C3 opsonin, secretary leukocyte protease inhibitor (SLPI), an antimicrobial peptide and B cells [142, 145, 146]. In addition to iron, zinc also plays an important role in regulation of *T. vaginalis* proteins. Total 27 proteins of *T. vaginalis* showed differential expression in the presence of zinc. Some of these proteins showed up-regulation while others showed down-regulation that was subsequently associated with modulation of some virulence properties *e.g.* cytotoxicity [147]. It was suggested that *T. vaginalis* can survive the highly dynamic vaginal milieu even during the extreme changes of menstruation. Recently, a study characterized a new virulence factor of *T. vaginalis,* named as TvMP50, a metalloproteinase mediated by zinc that contributes to cytotoxicity against DU145 prostatic cells [148].

#### T. vaginalis interaction with *Lactobacilli* in host VMB

Furthermore, adhesion of this parasite was shown to be increased by lactic acid [149]. Another study by Petrin et al. [125] showed that the parasite grows well at pH > 4.5, suggesting that normal acidic vaginal milieu, due to lactic acid, are important for the colonization of this parasite. After colonization, *T. vaginalis* modify the vaginal environment including fall in *Lactobacilli* and pH > 4.5, that supports its further growth and proliferation. This might be due to *T. vaginalis* mediated *Lactobacilli* phagocytosis that destabilize and challenges the host protective environment [150]. However, the exact mechanism behind this is still puzzling and has not been clearly elucidated. For this, better understanding of interaction between *T. vaginalis* and host VMB is needed. Recent studies have confirmed that vaginal microbiome changes during TV [151, 152]. *L. acidophilus* leads to increase in adherence of *T. vaginalis* to vaginal epithelial cells, during the early stages of infection [149]. As the number of *L. acidophilus* increases, the *T. vaginalis* become incapable of growing, while, vice versa occurs with decrease in number of *L. acidophilus* at the end of menses and during menopause, consequently leading to increase in TV symptoms [153]. This parasite has also been shown to have damaging effects on *L. acidophilus,* either by phagocytosis or via proteases [125]. Other than this, *L. gasseri* has been shown to inhibit adhesion of various *T. vaginalis* strains, while *L. plantarum* has been shown to enhance *T. vaginalis* adhesion by modifying their adhesive properties [154]. A study showed that TV transform the VMB of women by repressing bacteria associated with BV and increasing the growth of mycoplasma that may be the cause of its high prevalence found in women with intermediate BV [151]. A study based on pyrosequencing of barcoded 16S rRNA genes found an association of *T. vaginalis* with CST-IV of VMB including anaerobes *Sneathia*, *Parvimonas*, *Mycoplasma* and low levels of *Lactobacilli* [155].

#### Symbiotic associations of *T. vaginalis* and increased virulence

Moreover, a new species of *Mycoplamsa *i.e. “*Candidatus Mycoplasma girerdii”* primarily known as Mnola was found to be strongly associated with TV and was rarely found in healthy women [151, 156]. This species is shown to be symbiotically associated with *T. vaginalis* [151]. However, Fettweis et al. [156] suggest that “*Ca. M. girerdii*” is not an obligate symbiont of the parasite, contrary to *M. Hominis* and *M. Penetrans,* that are the obligatory symbionts and can penetrate as well as replicate inside *T. vaginalis* and upregulate energy production, cytolysis, growth rate and proinflammatory response of *T. vaginalis* [157, 158]. However, more studies are required to determine the symbiotic and synergistic relationship between “*Ca*. *M. girerdii*” and *T. vaginalis*. Additionally four intracellular dsRNA viruses were identified in *T. vaginalis* [159]. Recent studies have suggested that these viruses, along with other symbionts, enhance the expression of *T. vaginalis* virulence genes, consequently leading to increased adhesion to vaginal epithelial cells and cytotoxic activity against *Lactobacilli,* thus modify its growth rate and disease outcome [160, 161]. Other collaborative means by which different microbes including Mollicutes and dsRNA viruses, exibit symbiosis with *T. vaginalis* has been recently reviewed by Fichorova et al. [162]. Apart from these, *Leptothrix vaginalis*, a non-sporing, anaerobic, non-pathogenic, gram positive organism, is frequently found in association with *T. vaginalis*. However, its role in modulation of *T. vaginalis*‘s virulence remains elusive [163].

#### Unexplored role of genetic variability of *T. vaginalis*

Overall these studies confirm the potential role of TV in modulating vaginal milieu and importance of its interactions with VMB. However, similar to BV and VVC, asymptomatic cases of TV also exist [149, 164]. This suggests the presence of different virulent strains of *T. vaginalis,* as reported by some earlier studies [165, 166]. However, no studies based on whole genome comparison of different *T. vaginalis* strains are available. In fact, the first draft genome sequence of the *T. vaginalis* was reported in 2007 [167]. Although, effort was made by a study that has tried to identify epigenetic variations in conserved region of different *T. vaginalis* strains isolated from asymptomatic women using nested polymerase chain reaction-restriction fragment length polymorphism (PCR-RFLP) technique [164]. This suggests that more studies based on comparative genomics of *T. vaginalis* strains are needed. Exploration of host itself will also reveal the various indefinite factors and improve our understanding of TV pathogenesis that is still not clear.

### Dysbiosis leading to other dysbiosis (mixed infections) and foster infections

As mentioned above, milieu conditions of vagina during BV are the same as required for the transition of commensal *Candida* yeast to pathogenic hyphae and for the pathogenesis of TV. These similar conditions for pathogen prevalence are the rationale behind co-occurrence of many pathogens together leading to mixed infections and co-infections. A mixed infection is a condition in which there is more than one pathogen that is responsible for causing symptoms. Mostly these cases are of BV with VVC, BV with TV or combination of three [59]. This condition is mostly puzzled with cases of co-infections, where also more than one pathogen is present but only one pathogen is responsible for causing signs and symptoms. Additionally, if the three common VVI are left untreated, will not only effect the female reproductive health but may also result in many foster infections/diseases and adverse pregnancy outcomes. These vaginal infections effect reproductive health leading to infertility because of fallopian tube occlusion [168]. The most common examples of foster infections include bacterial and viral sexually transmitted infections (STIs). Other than *T. vaginalis*, *C. trachomatis* and *Neisseria Gonorrhoeae* are the most common sexually transmitted bacteria that can occur as advance infections, if previous infections left untreated [169]. The best depiction of it is, BV leading to foster *C. trachomatis* infection. Tryptophan is an important component of *C. trachomatis* nutrition and thus relies on pool of host tryptophan for establishment and pathogenesis. This advantage is taken by host by producing IFN-γ that activates the human indoleamine-2,3-dioxygenase (IDO1) enzyme, for the degradation of tryptophan, eventually leading to tryptophan deficiency and *C. trachomatis* clearance. However, indole produced by anaerobes present in VMB of BV (also in CST-IV) is used by *C. trachomatis* as an alternative substrate for tryptophan synthesis by their own tryptophan synthase enzyme, thus allowing its establishment and pathogenesis by avoiding host immune responses [170].

Moreover, *Lactobacilli*, the dominant flora of healthy VMB, have been shown to reduced risk of sexually transmitted *N. Gonorrhoeae* infection [7, 171]. *L. jensenii* and *L. gasseri,* have been shown to inhibit both adherence and invasion of *N. Gonorrhoeae*, the two main steps required for establishment of an infection. Furthermore, these *Lactobacilli* have also been shown to act as post exposure prophylactic, because of their ability to displace already adhered gonococci from vaginal epithelial cells [171]. However, absence of *Lactobacilli*, during common vaginal infections may lead to *N. Gonorrhoeae* infection by allowing its establishment and pathogenesis. In addition, VMB during VVIs mainly BV, were found to be associated with many viral STIs including HIV, human papilloma virus (HPV), HSV and precancerous cervical lesions [reviewed in 172]. Additionally, dysbiosis during vaginal infections also leads to pelvic inflammatory disease [173]. Though, the data is not entirely consistent with these associations, but all are directed to increased anaerobic diversity, leading to foster infections. Recently, a study suggested the increased risk of HIV acquisition with RBV (87). Not only this, studies have linked the ascending of vaginal infections to mother womb leading to adverse pregnancy outcomes [174, 175]. BV was found to be associated with increased risks of preterm birth, early pregnancy loss; spontaneous abortion, low birth weight, increased neonatal morbidity and higher rates of postpartum endometritis [168, 176, 177]. Thus, treatment of vaginal infections is mandatory for female reproductive health and to prevent adverse foster infections leading to morbidity. Finding the key reason behind these infections is important to develop the new preventive strategies.

### Controversies

“A VMB dominated with *Lactobacilli* is healthier than a diverse VMB” was indeed a widely recognized dogma at an earlier time, due to the health benefits of *Lactobacilli* and dominance in approximately 70% of healthy women. However, this statement seemed to be challenged by two disputes uncovered by high throughput sequencing approaches. First, the term beneficial and protective is not fully applicable on all the *Lactobacillus* species. For example, *L. iners* is considered to be pathogenic due to its pore forming ability in vaginal epithelial cells, presence of virulence factors such as inerolysin and cytolysin, absence of protective factors like H_2_O_2_, D(–) lactic acid, very less production of L(+) lactic acid and due to its reported associations with preterm birth as well as BV [178–180]. Similarity, *L. jensenii* produces only D(–) lactic acid and both *L. gasseri* and *L. iners* were found to be associated with VMB instability in pregnant women [178, 179]. It is only *L. crispatus* that is so far consistently been linked with good vaginal health, VMB stability and lower risk of developing VVI [181]. This suggests the species specific effects of *Lactobacilli* on host.

Moreover, as aforementioned, there is increased evidence regarding the presence of diverse VMB in 20–30% of healthy women that are also designated as asymptomatic [8]. Some studies have suggested ethnic and regional differences in stipulations of what is actually healthy, as high percentage of these asymptomatic cases belongs to Black and Hispanic women [8, 182]. But again the biggest puzzle is, small but definite proportion of these asymptomatic cases is also found in women of other ethnic groups having high proportion of CSTs dominated by *Lactobacilli* [183]. Moreover, a recent study using both culture dependent and independent approaches claimed of no ethnic difference in VMB of white and black asymptomatic women [184]. In addition, other studies have suggested that diverse VMB in asymptomatic women is healthy, as these species e.g. *Atopobium*, *Megasphaera* and *Leptotrichia* are capable of producing lactic acid and other antimicrobial substances [8, 9]. This is again mystifying due to the presence of these species in BV women and their contribution in increasing pH and BV pathogenesis. This is due to these controversies, that in spite of doing decades of research, we are not being able to find a single pathogen or etiological agent responsible for causing RVVI. Since, these microbes could not satisfy the Koch’s postulates, according to which the causative agent is essential for causing disease and should not be present in healthy population [185]. Owing to these reasons, studies over the past decade on VMB have complicated the definition of vaginal health and vaginal infections with no definite conclusion.

## Conclusions

**Fig. 5:**
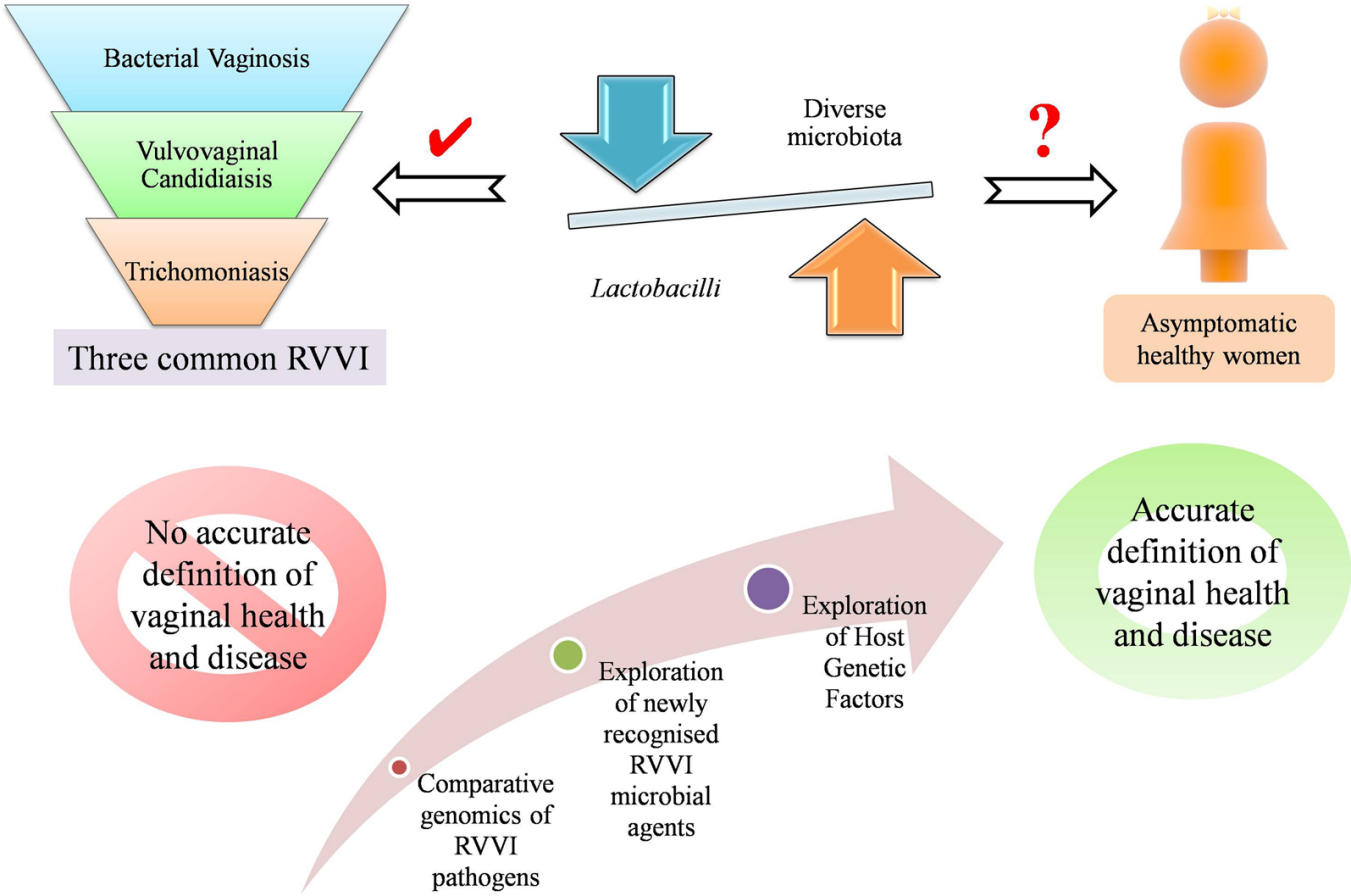
Controversial nature of vaginal microbiota in health as well as disease and future directions

Though, culture-independent methods have greatly unfolded the mystery regarding vaginal bacterial microbiome, there are only a few studies regarding the composition and diversity of vaginal mycobiome and different *Trichomonas vaginalis* strains. This scenario suggests a need of further studies based on comparative genomics of RVVI pathogens to improve our perceptive of RVVI pathogenesis that is still not clear (Fig. 5). Moreover, the list of possible agents continues to expand, further studies on newly recognised microbial agents, may reveal answers to the controversies. Conversely, VMB of women could be an answer but it is not enough to just look at the microbiology. We have to look at the woman itself, as VMB which is fine for one woman may be troublesome for others. These differences in women’s response to the same VMB may be determined by a permutation of behavioural, cultural, genetic and various other anonymous factors. Exploration of these factors may lead to proper definition of vaginal health and disease.

## Data Availability

The data that support the findings of this study are available from the corresponding author upon reasonable request.

## Abbreviations

RVVI: Recurrent vulvovaginal infections
HIV: Human immunodeficiency virus
WHO: World Health Organization
CDC: Centres for diseases control
VVC: Vulvovaginal Candidiaisis
TV: Trichomoniasis
BV: Bacterial vaginosis
VMB: Vaginal microbiota
CSTs: Community state types
HSV-2: Herpes simplex virus type 2
IL-1RA: Interleukin-1 receptor antagonist
TNFα: Tumor necrosis factor α
RANTES: Regulated on activation, normal T cell expressed and secreted
MIP3α: Macrophage inflammatory protein-3 alpha
TGF-β: Transforming growth factor beta
Th17: T helper 17
cAMP: Cyclic adenosine monophosphate
O_2_: Oxygen
HIV-1: HIV type-1
HCl: Hydrochloric acid
NAC: Non-albicans Candida
SP-A: Surfactant protein A
VVI: Vulvovaginal infections
RBV: Recurrent BV
RVVC: Recurrent VVC
RTV: Recurrent TV
BVAB1: Bacterial vaginosis-associated bacteria type 1
AV: Aerobic vaginitis
AlS3: Agglutinin-like sequence protein
SAP: Secreted aspartic proteases
Hwp: Hyphal wall protein
SNPs: Single nucleotide polymorphisms
CNVs: Copy number variations
PFOR: Pyruvate ferredoxin oxidoreductase
Ig: Antibodies
SLPI: Secretary leukocyte protease inhibitor
PBMCs: Peripheral blood mononuclear cells
RBCs: Red blood cells
PCR–RFLP: Polymerase chain reaction-restriction fragment length polymorphism
STIs: Sexually transmitted infections
IDO1: Indoleamine-2,3-dioxygenase
HPV: Human papilloma virus
H_2_O_2_: Hydrogen peroxide
